# Correction: The mechanism of the molecular CISS effect in chiral nano-junctions

**DOI:** 10.1039/d4sc90193b

**Published:** 2024-10-04

**Authors:** Thi Ngoc Ha Nguyen, Georgeta Salvan, Olav Hellwig, Yossi Paltiel, Lech Tomasz Baczewski, Christoph Tegenkamp

**Affiliations:** a Solid Surface Analysis, Institute of Physics, Chemnitz University of Technology 09126 Chemnitz Germany christoph.tegenkamp@physik.tu-chemnitz.de; b Semiconductor Physics, Institute of Physics, Chemnitz University of Technology 09126 Chemnitz Germany; c Functional Magnetic Materials, Institute of Physics, Chemnitz University of Technology 09126 Chemnitz Germany; d Institute of Ion Beam Physics and Materials Research, Helmholtz-Zentrum Dresden-Rossendorf 01328 Dresden Germany; e Department of Applied Physics, Hebrew University of Jerusalem 91904 Jerusalem Israel; f Center for Nanoscience and Nanotechnology, Hebrew University of Jerusalem 91904 Jerusalem Israel; g Institute of Physics, Polish Academy of Sciences 02-668 Warszawa Poland

## Abstract

Correction for ‘The mechanism of the molecular CISS effect in chiral nano-junctions’ by Thi Ngoc Ha Nguyen *et al.*, *Chem. Sci.*, 2024, **15**, 14905–14912, https://doi.org/10.1039/D4SC04435E

In the original article, the name of one of the authors, Lech Tomasz Baczewski, was displayed incorrectly. The correct presentation of this name is as shown above.

The Royal Society of Chemistry apologises for these errors and any consequent inconvenience to authors and readers.

